# Supplementary material for: A toolkit for planning and implementing acute febrile illness (AFI) surveillance
Source: PLOS Glob Public Health. 2024 Apr 18;4(4):e0003115. doi: 10.1371/journal.pgph.0003115 (PMC11025857; doi:10.1371/journal.pgph.0003115)
Supplement: S2 File — (DOCX) [file pgph.0003115.s002.docx]

**Members of working group for development of AFI surveillance toolkit**

- Adam L. Cohen, U.S. Centers for Disease Control and Prevention, Atlanta, GA
- Amanda Balish, U.S. Centers for Disease Control and Prevention, Atlanta, GA
- Carl Kinkade, U.S. Centers for Disease Control and Prevention, Atlanta, GA
- Casey Siesel, U.S. Centers for Disease Control and Prevention, Atlanta, GA
- Daniel Martin, U.S. Centers for Disease Control and Prevention, Atlanta, GA
- David Shih, U.S. Centers for Disease Control and Prevention, Atlanta, GA
- Jonathan Bryant, U.S. Centers for Disease Control and Prevention, Atlanta, GA
- Lila Rahalison, U.S. Centers for Disease Control and Prevention, Atlanta, GA
- Lilit Kazazian, U.S. Centers for Disease Control and Prevention, Atlanta, GA
- Madeline Farron, U.S. Centers for Disease Control and Prevention, Atlanta, GA
- Matthew Mikoleit, U.S. Centers for Disease Control and Prevention, Atlanta, GA
- Michael Park, U.S. Centers for Disease Control and Prevention, Atlanta, GA
- Nicolas Schaad, U.S. Centers for Disease Control and Prevention, Atlanta, GA
- Olga Henao, U.S. Centers for Disease Control and Prevention, Atlanta, GA
- Pawan Angra, U.S. Centers for Disease Control and Prevention, Atlanta, GA
- Rachel Silver, U.S. Centers for Disease Control and Prevention, Atlanta, GA
- Tori Hicks, U.S. Centers for Disease Control and Prevention, Atlanta, GA

**Technical reviewers of AFI surveillance toolkit**

- Anik Palit, icddr,b, Dhaka, Bangladesh
- Christopher Murrill, U.S. Centers for Disease Control and Prevention, Atlanta, GA
- Ekaterine Khmaladze, National Center for Disease Control and Public Health, Tbilisi, Georgia
- Elijah Paa Edu-Quansah, African Field Epidemiology Network, Monrovia, Liberia
- Eric Nilles, Brigham and Women’s Hospital, Boston, MA
- Fahmida Chowdhury, icddr,b, Dhaka, Bangladesh
- Gerald Pellegrini, U.S. Centers for Disease Control and Prevention, Atlanta, GA
- Mahabub Anwar, U.S. Centers for Disease Control and Prevention, Dhaka, Bangladesh
- María Beatriz López Castellanos, U.S. Centers for Disease Control and Prevention, Guatemala City, Guatemala
- Mohammed Ziaur Rahman, icddr,b, Dhaka, Bangladesh
- Ornuma Sangwichian, Thailand Ministry of Public Health–U.S. Centers for Disease Control and Prevention Collaboration, Nonthaburi, Thailand
- Pongpun Sawatwong, Thailand Ministry of Public Health–U.S. Centers for Disease Control and Prevention Collaboration, Nonthaburi, Thailand
- Saithip Bhengsri, Thailand Ministry of Public Health–U.S. Centers for Disease Control and Prevention Collaboration, Nonthaburi, Thailand
- Tanzir Ahmed Shuvo, icddr,b, Dhaka, Bangladesh
- Tinatin Kuchuloria, U.S. Centers for Disease Control and Prevention, Tbilisi, Georgia
