# Supplementary material for: A toolkit for planning and implementing acute febrile illness (AFI) surveillance
Source: PLOS Glob Public Health. 2024 Apr 18;4(4):e0003115. doi: 10.1371/journal.pgph.0003115 (PMC11025857; doi:10.1371/journal.pgph.0003115)
Supplement: S3 File — (DOCX) [file pgph.0003115.s003.docx]

| **Project Overview** | | | | | |
| --- | --- | --- | --- | --- | --- |
| **Primary objective**   - Protocol Section 2.2.1 | *What do you primarily want to accomplish through this AFI surveillance project?*  Identify priority AFI etiologies (if priority etiologies are unknown)  Monitor priority AFI etiologies (if priority etiologies are known or will be identified) | | | | |
| **Secondary objectives**   - Protocol Section 2.2.2 | *What additional objectives could be accomplished with this surveillance project?* *Select all that apply:*  To describe the epidemiological characteristics and risk factors of AFI cases at selected sentinel sites in _________________[COUNTRY/REGION], using basic demographics such as patient age, sex, and geographic area  To improve epidemiologic and laboratory capacity to identify, monitor, and assess pathogens of potential public health importance  To determine public health priorities, guide programmatic improvement for patient management, and inform resource allocations  To establish a biobank of samples that can be used to meet future public health needs  To evaluate the performance of different specimen types and diagnostic technologies for the diagnosis of etiologies of AFI  To guide and measure impact of public health interventions  Other (specify): __________________________________________________________ | | | | |
| **Surveillance timeframe**   - Protocol Section 3.1 | *Start (month and year)*: | | | *End (month and year) [if applicable]*: | |
| **Surveillance setting and population**   - Protocol Section 3.2 | *What is the surveillance setting?*  Urban area  Rural area  Combination | | | | |
|  | *List the geographic area(s) (e.g., cities, regions) where the surveillance will take place:* | | | | |
|  | *From which type(s) of sentinel sites will patients be recruited? Select all that apply:*  Community health facility/clinic; initial number of sites: ______  Hospital – inpatient; initial number of sites: ______  Hospital – outpatient; initial number of sites: ______  Other (specify): ___________________________; initial number of sites: ______ | | | | |
|  | *Describe the population(s) served at the sites:* | | | | |
|  | *Describe any plans to expand over time:* | | | | |
| **Patient Enrollment** | | | | | |
| **Case definition**   - Protocol Section 3.3.1 | **Inclusion Criteria** | | | | |
|  | *Documented fever using varying measurement sites (select all that apply and specify minimum temperature):*  Axillary: °C  Oral: °C  Rectal: °C  Tympanic: °C  Other ________: °C | *Fever onset date:*  Onset within previous _____ days | | | *History of fever:*  Yes, only measured history of fever  Yes, including subjective history of fever  No |
|  | *Note: Sites may enroll patients with temperatures < 38°C (e.g., ≥ 37.5°C, ≥ 37°C) but should record the measured temperature value of each patient.* | *Note: Sites may enroll patients with fever for > 7 days (e.g., onset within the past 14 days) but should record the date of fever onset of each patient.* | | | *Note: Sites may enroll patients with a reported history of unmeasured fever but should be able to distinguish these patients from those with measured fever.* |
|  | *Which age groups will be eligible for the surveillance? Select all that apply:*  Neonates (<1 month)  Older children and adolescents (5-19 years)  Infants (1-23 months)  Adults (40-59 years)  Children (2-5 years)  Elderly (>=60 years) | | | | |
|  | *Is the surveillance targeted towards a demographic group with specific risk factors?*  Yes (explain): ________________________________________  No | | | | |
|  | **Exclusion Criteria** | | | | |
|  | *Core exclusion criteria:*  Refusal or inability to consent or assent to participation  Presenting with confirmed cause of fever  Chief complaint is injury or trauma | | | | |
|  | *Optional exclusion criteria:*  Evidence of syndromes outside the scope of the surveillance (specify):  __________________________________________________________________________  Enrolled in this AFI surveillance within the past ________ weeks / months / years  Other (specify): ___________________________________________________________ | | | | |
| **Control enrollment**   - Protocol Section 3.3.4 | *Will controls be enrolled in this study alongside cases?*  Yes  No | | | | |
|  | *If yes, where will they be enrolled?*  ☐ Households in catchment areas  Health facilities  Other (specify): ___________________________________________________________ | | | | |
| **Specimen Collection and Laboratory Procedures** | | | | | |
| **Specimen collection**   - Protocol Section 3.4.1 | *Which specimens will be collected from patients? Select all that apply:*  ☐ Blood (acute)  Blood (convalescent)  Urine  Stool  Sputum  NP/OP swabs  Other (specify):___________________________________ | | | | |
|  | *Will selected specimens be collected from* ***all*** *enrolled patients?*  Yes  No (specify sampling method):_______________________________________________ | | | | |
| **Laboratory testing**   - Protocol Section 3.4.3 | *Select all testing methods/platforms to be used to detect pathogens of interest:* | | *List the pathogens to be investigated by each method/platform:* | | |
|  | ☐ Multi-pathogen PCR (e.g., TaqMan Array Card, BioFire) | |  | | |
|  | Single-pathogen PCR | |  | | |
|  | Culture | |  | | |
|  | Serology (e.g., IgG, IgM, IgA) | |  | | |
|  | Microscopy | |  | | |
|  | Rapid diagnostic testing (RDT) | |  | | |
|  | Other (specify): | |  | | |
| **Data Management** | | | | | |
| **Data integration with existing systems**   - Protocol Section 4.1 | *Will surveillance data integrate with an existing national surveillance system (e.g., LIMS, DHIS-2, EIDSS)? If yes, describe:* | | | | |
| **Data collection platform**   - Protocol Section 4.1 | *How will surveillance data be collected? Select all that apply:*  ☐ Paper data collection  Real-time electronic data collection (e.g., tablet, mobile device)  *If electronic, which platform will be used?*  REDCap  EpiInfo  Other (specify):___________________________________ | | | | |
