# Supplementary material for: A toolkit for planning and implementing acute febrile illness (AFI) surveillance
Source: PLOS Glob Public Health. 2024 Apr 18;4(4):e0003115. doi: 10.1371/journal.pgph.0003115 (PMC11025857; doi:10.1371/journal.pgph.0003115)
Supplement: S4 File — (DOCX) [file pgph.0003115.s004.docx]

**Generic Protocol for Surveillance of**

**Acute Febrile Illness**

**Table of Contents**

[**List of Acronyms** 4](#_Toc86994841)

[**1.** **Surveillance Overview** 5](#_Toc86994842)

[**1.1** **Title** 5](#_Toc86994843)

[**1.2** **Summary** 5](#_Toc86994844)

[**1.3** **Investigators and Technical Advisors** 5](#_Toc86994845)

[1.3.1 Principal Investigator 5](#_Toc86994846)

[1.3.2 Co-Investigators 5](#_Toc86994847)

[1.3.3 Technical Advisors 5](#_Toc86994848)

[**1.4** **Roles and Responsibilities** 5](#_Toc86994849)

[1.4.1 Principal Investigator 5](#_Toc86994850)

[1.4.2 Co-Investigators 5](#_Toc86994851)

[1.4.3 Technical Advisors 5](#_Toc86994852)

[1.4.4 Implementing Partner 5](#_Toc86994853)

[1.4.5 Other Partners 5](#_Toc86994854)

[**1.5** **Funding Mechanism** 5](#_Toc86994855)

[**2.** **Introduction** 6](#_Toc86994856)

[**2.1** **Background and Justification** 6](#_Toc86994857)

[**2.2** **Objectives** 6](#_Toc86994858)

[2.2.1 Primary Objective 6](#_Toc86994859)

[2.2.2 Secondary Objectives 6](#_Toc86994860)

[**3.** **Methodology** 7](#_Toc86994861)

[**3.1** **Design** 7](#_Toc86994862)

[**3.2** **Setting and Population** 7](#_Toc86994863)

[**3.3** **Patient Screening, Sampling, and Enrollment** 8](#_Toc86994864)

[3.3.1 Patient Screening and Case Definition 8](#_Toc86994865)

[3.3.2 Sampling Framework 9](#_Toc86994866)

[3.3.3 Patient Enrollment and Data Collection 9](#_Toc86994867)

[3.3.4 Expected Enrollment 10](#_Toc86994868)

[**3.4** **Specimen Collection and Laboratory Procedures** 10](#_Toc86994869)

[3.4.1 Specimen Collection 10](#_Toc86994870)

[3.4.2 Specimen Storage and Transport 12](#_Toc86994871)

[3.4.3 Specimen Processing and Testing 12](#_Toc86994872)

[3.4.4 Specimen Archiving 13](#_Toc86994873)

[**4.** **Data Management and Analysis** 13](#_Toc86994874)

[**4.1** **Data Management** 13](#_Toc86994875)

[**4.2** **Data Cleaning and Analysis** 14](#_Toc86994876)

[**4.3** **Data Sharing** 15](#_Toc86994877)

[**5.** **Ethical Considerations** 15](#_Toc86994878)

[**5.1** **Informed Consent/Assent** 15](#_Toc86994879)

[**5.2** **Risks** 16](#_Toc86994880)

[**5.3** **Benefits** 16](#_Toc86994881)

[**6.** **Timeline** 17](#_Toc86994882)

[**6.1** **Future Protocol Adaptation** 17](#_Toc86994883)

# **List of Acronyms**

*Update the below list after revision of the protocol.*

AFI Acute febrile illness

CRF Case Report Form

ELISA Enzyme-linked immunosorbent assay

IFA Immunofluorescence assay

IRB Institutional Review Board

NP/OP Nasopharyngeal/oropharyngeal

PCR Polymerase chain reaction

PI Principal Investigator

RDT Rapid diagnostic test

# **Surveillance Overview**

## **Title**

Surveillance of Acute Febrile Illness (AFI) in [COUNTRY/REGION]

## **Summary**

*Summarize the overarching goals and key activities once the rest of the protocol has been finalized.*

## **Investigators and Technical Advisors**

### Principal Investigator

- [NAME, TITLE, INSTITUTION AND CONTACT INFORMATION]

### Co-Investigators

- [NAME, TITLE, INSTITUTION AND CONTACT INFORMATION]
- [ETC…]

### Technical Advisors

- [NAME, TITLE, INSTITUTION AND CONTACT INFORMATION]
- [ETC…]

*Additional headings in Section 1.3 could include “Co-Principal Investigator(s)”, “Project Leader(s)”, and any other project-specific entities. Delineate the roles and responsibilities of each entity in Section 1.4.*

## **Roles and Responsibilities**

### Principal Investigator

*Provide a brief description of Principal Investigator role and responsibilities.*

### Co-Investigators

*Provide a brief description of Co-Investigator roles and responsibilities.*

### Technical Advisors

*Provide a brief description of Technical Advisor roles and responsibilities.*

### Implementing Partner

*Update heading with name of implementing partner and provide a brief description of its role and responsibilities. The role of the implementing partner may include identifying sites, enrolling patients, laboratory testing, and liaising with the Ministry of Health.*

### Other Partners

*Create headings for each additional partner who provides support for implementation. Provide a brief description of their roles and responsibilities.*

## **Funding Mechanism**

This surveillance is funded by [SOURCE OF FUNDS].

# **Introduction**

## **Background and Justification**

Acute Febrile Illness (AFI) is a common manifestation of a wide spectrum of infectious diseases, some treatable and preventable. The etiologies of AFI (which include bacteria, fungi, viruses, and parasites) vary geographically and seasonally and can change over time due to factors such as the use of vaccines, environmental changes, sanitation, and economic development. Given the similarity of AFI clinical presentations and diversity of potential etiologies, diagnosis is a major challenge in settings with limited laboratory diagnostic capability and capacity. As a result, clinicians often manage AFI cases empirically, leading to misdiagnosis, poor treatment outcomes, and flawed estimates of baseline disease prevalence.

Understanding the etiologies of AFI in a population or region can guide case management and help to identify and strengthen gaps in surveillance systems, laboratory capacity, and public health preventative measures. Because emerging and re-emerging infectious diseases often present with non-specific symptoms, effective AFI diagnosis and surveillance is also critical to achieving global health security.

*Provide additional country-specific background information, including but not limited to:*

*Summary of AFI burden in the country and region*

*Description of existing AFI and/or related surveillance systems (e.g., influenza-like illness and severe acute respiratory illness)*

*Current knowledge on underlying AFI etiologies*

*Remaining knowledge gaps to be addressed by current surveillance*

## **Objectives**

### Primary Objective

To [IDENTIFY AND/OR MONITOR] priority AFI etiologies among [TARGET POPULATION] presenting to [TYPE(S) OF SENTINEL SITES] in [COUNTRY/REGION].

­

**Considerations for Adaptation**

Target population: The target population may include specific demographic groups (e.g., children, agricultural workers), depending on surveillance priorities and the feasibility of implementation (see Section 3.3.1 for details).

Sentinel sites: The selection of sentinel sites is often limited by resources and capacity, but additional considerations include geographic and demographic representativeness (see Sections 3.1­–3.2 for details). _­_­­­­­

### Secondary Objectives

*Secondary objectives will vary based on surveillance goals and limitations. Examples include:*

- *To describe the epidemiological characteristics and risk factors of AFI cases at selected sentinel sites in [COUNTRY/REGION], using basic demographics such as patient age, sex, and geographic area*
- *To improve surveillance and laboratory capacity to identify, monitor, and assess pathogens of potential public health importance*
- *To determine public health priorities, guide programmatic improvement for patient management, and inform resource allocations*
- *To establish a biobank of samples that can be used to meet future public health needs*
- *To evaluate the performance of different specimen types and diagnostic technologies for the diagnosis of etiologies of AFI*
- *To guide and measure impact of public health interventions*

**Considerations for Adaptation**

Secondary objectives: It is important to consider the trade-offs inherent to the selection of these objectives – a greater focus on one objective often leaves less room to pursue others. For example, establishing a biobank is a very resource-intensive endeavor that requires procuring and ensuring maintenance of freezers, as well as considering biosafety and biosecurity guidelines. While biobank development is a worthwhile objective, it may not be a priority for all countries conducting AFI surveillance.

# **Methodology**

## **Design**

This project is an active, prospective, case-based sentinel surveillance of AFI with laboratory confirmation of each case in [COUNTRY/REGION]. The surveillance will be launched in [MONTH AND YEAR] and data collection will continue for [NUMBER OF MONTHS/YEARS] in [NUMBER OF SENTINEL SITES].

*Describe the phases of implementation, including any plans for expanding to more sites over time. Indicate whether this project is research or routine public health surveillance. Provide additional details as necessary to describe the overall surveillance design, including plans for integration with other surveillance systems.*

**Considerations for Adaptation**

Sentinel vs. population-based surveillance: Because the catchment area of sentinel sites is not usually well-defined, it can be difficult to use sentinel surveillance to estimate AFI incidence. However, including every sentinel site in a distinct geographic area approximates population-based surveillance if denominators are known. This approach is usually more costly but allows for more generalizable surveillance data. Note that data on healthcare seeking is necessary to estimate incidence rates among the general population.

## **Setting and Population**

*Describe the sentinel sites and geographic area where the surveillance will be implemented. Include:*

- *Names and location(s) of sites*
- *Description of geographic area and population(s) served at sites*
- *Type(s) of sites (e.g., community health facility, hospital, clinic)*
- *Relevant context, including previous and existing surveillance activities at sites*
- *Reasons for site selection*
- *Sample transport between recruitment and laboratory testing site(s), if applicable*

**Considerations for Adaptation**

Sentinel sites: The selection of sentinel sites requires balancing a variety of considerations. Selection criteria may include geographic representativeness, patient population and volume, known disease burden, degree of urbanization, government prioritization, opportunities to leverage existing platforms, laboratory infrastructure, specimen transport networks, and logistical concerns (e.g., distance, adequate personnel, security). For practical reasons, many countries choose to begin with a limited number of sentinel sites in a “pilot” phase before expanding to more sites.

## **Patient Screening, Sampling, and Enrollment**

### Patient Screening and Case Definition

Site surveillance staff will conduct [FREQUENCY (e.g., daily, weekly)] screening of [PROPORTION OF PATIENTS (e.g., all patients, every *n*th patient)] seen in [OUTPATIENT, INPATIENT, OR BOTH] areas at the selected sites. Patient screening will occur according to the below criteria using the AFI Screening Form (Appendix [X]).

Inclusion criteria

*CORE:*

- Measured [AXILLARY, TYMPANIC, ORAL, AND/OR RECTAL] temperature ≥ 38°C* with onset within the past 7 days** in patients [AGE RANGE OR OTHER DEMOGRAPHIC CRITERIA] [ADMITTED TO/TREATED AT SPECIFIC FACILITIES]

*OPTIONAL:*

- *History of new fever (unmeasured)*** with onset within previous 7 days***

**Sites may enroll patients with temperatures < 38°C (e.g., ≥ 37.5°C, ≥ 37°C) but should record the measured temperature value of each patient.*

***Sites may enroll patients with fever for > 7 days (e.g., onset within the past 14 days) but should record the date of fever onset of each patient.*

****Sites may enroll patients with a reported history of unmeasured fever but should be able to distinguish these patients from those with measured fever.*

Exclusion criteria

*CORE:*

- Refusal or inability to consent or assent to participation
- Presenting with confirmed cause of fever
- Chief complaint is injury or trauma
- Enrolled in this AFI surveillance within the past year

*OPTIONAL:*

- *Specify any additional syndromes (e.g., respiratory syndromes, gastrointestinal syndromes, rash) outside the scope of the surveillance.*
- *Exclude patients enrolled in this AFI surveillance within a specified period of time (e.g., the last year).*

**Considerations for Adaptation**

Temperature threshold: Temperature values may vary by measurement site (e.g., axillary, tympanic, oral, rectal). If desired, different temperature thresholds may be selected for each measurement site based on the range of normal values identified in the literature (e.g., [this 2019 systematic review](https://doi.org/10.1093/ofid/ofz032)). In general, a lower temperature threshold is more sensitive (i.e., better at capturing all illnesses) but less specific (i.e., capturing non-infectious etiologies). If sites enroll patients with temperatures < 38°C, the measured temperature value must be recorded to allow subset analysis of patients with temperatures ≥ 38°C.

History of fever: The chances of pathogen detection are typically highest within 7 days of fever onset, but sites may choose to enroll patients with a longer fever duration to increase sensitivity. Reported history of fever, while less reliable than measured fever, may be important for capturing patients who may not present with fever due to antipyretic use before arrival. If sites enroll patients with fever for > 7 days and/or those with an unmeasured history of fever, these data must be recorded to allow subset analysis of patients with measured fever that started within the past 7 days.

Additional eligibility criteria: Including all febrile individuals presenting to sentinel sites might be challenging or not feasible to implement. Individuals from specific demographic groups (e.g., age, sex, occupation) or with evidence of specific syndromes (e.g., urinary tract infection, otitis media) may be included or excluded depending on context-specific considerations. Patients previously enrolled in this AFI surveillance may either be re-enrolled (assuming enough time has passed to be infected with a new pathogen) or excluded to represent a wider population of patients.

### Sampling Framework

*Describe the sampling framework used to enroll eligible individuals. Options for systematic sampling include:*

- *Enrolling all eligible individuals*
- *Enrolling every* n*th individual meeting eligibility criteria (where n is an integer > 1)*
- *Recruiting on specified days of the week*
- *Randomly selecting from among eligible individuals*

*If systematic sampling is infeasible – for example, if a cap exists on maximum number of enrollees per week – determine which characteristics (e.g., age, site, time of year) will be disproportionally represented in the sample of enrolled patients. Describe how this bias will be accounted for (e.g., by collecting data on eligible non-participants and weighting accordingly).*

**Considerations for Adaptation**

Sampling framework: Enrolling all eligible individuals generally allows for the least biased estimates. However, a sampling scheme may be warranted based on anticipated AFI patient volume, available resources, and staffing constraints. The sample should be as random as possible and account for any irregularities in patient presentation. If the sampling scheme will result in systematic misrepresentation of certain patient characteristics (e.g., age, site, time of year), then combining patient data across these characteristics will lead to bias. Sampling weights are an imperfect way to correct for this bias based on the characteristics of eligible non-participants to make estimates based on pooled patient data (e.g., overall AFI etiology prevalence, proportion of cases by age group).

### Patient Enrollment and Data Collection

All eligible sampled patients who provide informed consent or assent for participation (detailed in section 5.1) will be enrolled and assigned a unique identification number to be used on all data forms and specimen labels.

*Describe how unique identification numbers will be generated.*

[DESIGNATED STAFF] will administer the [ELECTRONIC OR PAPER-BASED] Case Report Form (Appendix [X]) to all enrolled patients. The Case Report Form will collect basic demographic (e.g., age, sex) and clinical (e.g., symptoms, signs, treatment prior to enrollment) information from all enrolled cases.

*If applicable, describe any additional sections of the Case Report Form, such as epidemiological risk factors, results of relevant tests performed during routine clinical care, and outcomes (see setting-specific questions in generic data dictionary). Ascertaining outcomes, especially discharge and death, can be difficult and require detailed process description in this section. Describe any follow-up data collection forms and procedures.*

**Considerations for Adaptation**

Paper-based vs. electronic data collection: Real-time electronic data collection encourages enhanced data quality, faster turnaround times for data analysis and dissemination, and long-term reductions in labor costs. Considerations for implementing a real-time electronic data collection platform include the availability of electrical and/or mobile connectivity, informatics support, and the remoteness of collection sites. Sites may consider starting with paper forms and transitioning to electronic capture.

### Expected Enrollment

Annual enrollment is expected to be [NUMBER OF CASES (AND CONTROLS, if applicable) PER SITE].

*If not enrolling controls and not conducting hypothesis testing:* As this activity does not involve the estimation of any population-based parameters or *a priori* hypothesis testing, no sample size calculation is required.

**Considerations for Adaptation**

Expected enrollment: Estimates of expected enrollment over the course of the surveillance period may be based on existing surveillance data, facility registers, or patient records. If not readily available, primary data on hospital visits from AFI patients may be collected.

Controls: Enrollment of asymptomatic controls, in addition to AFI patients, allows characterization of the baseline non-AFI population. When rigorously sampled and recruited, such a case-control methodology can allow estimation of risk factors for AFI and the etiologic fraction attributed to the pathogens of interest. However, hospital or clinic-based controls rarely represent the source population from which AFI cases arise. Unless resources exist to recruit a representative sample, sampling controls might not yield valid etiologic fraction estimates.

## **Specimen Collection and Laboratory Procedures**

### Specimen Collection

After patient enrollment, [DESIGNATED STAFF] will collect blood [AND ADDITIONAL SAMPLE TYPES (if applicable)] from [SPECIFIED PATIENTS (e.g., all patients, those with specific symptoms)]. Each specimen will be labeled with the unique patient identifier. [DESIGNATED STAFF] will also complete the [ELECTRONIC OR PAPER-BASED] Specimen Requisition Form (Appendix [X]) for each patient at the time of specimen collection.

Blood: Whole blood will be collected aseptically into a [SPECIFIC TUBE AND/OR CULTURE BOTTLE] by venipuncture using [DESCRIBE COLLECTION TECHNIQUE]. *Describe the volume of blood to be collected from adults and children and the proportion to be used for molecular testing, serological testing, and/or culture.*

*If collecting serum/plasma samples:* Blood will be centrifuged at [LOCATION (ideally, the testing site)] to separate the [SERUM OR PLASMA] for serological testing and avoid hemolysis. The resulting [SPECIFIED VOLUME] of [SERUM OR PLASMA] will be drawn off and placed in a sterile, leak-proof, screw capped tube.

*If collecting convalescent samples:* [SPECIFIED PATIENTS] will be asked to return for a convalescent sample [NUMBER OF DAYS] after the initial sample. [SPECIFIED VOLUME] of blood will be drawn into a serum separator tube [(RED TOP OR “TIGER TOP”)]. At the time of convalescent specimen collection, an [ELECTRONIC OR PAPER-BASED] follow-up data collection form will be administered. *Describe the process of reminding patients of their follow-up appointments and whether they will be compensated for their time and transportation costs. Include any follow-up data collection forms in the appendix.*

*Create new sections to describe the collection of any additional specimens, which might include nasopharyngeal (NP) or oropharyngeal (OP) swabs, urine, stool, sputum, tissue, or cerebrospinal fluid (CSF). Refer to established protocols such as the* [*Manual for Identification and Antimicrobial Susceptibility Testing (World Health Organization, 2003)*](https://apps.who.int/iris/bitstream/handle/10665/68554/WHO_CDS_CSR_RMD_2003.6_%28appendices%29.pdf?sequence=2&isAllowed=y) *for collection procedures for NP/OP swabs (p. 251), stool (p. 275), or CSF (p. 223). Note that CSF collection must only be performed by qualified and trained medical staff.*

*The protocol should specify (or refer to) procedures sufficient to ensure appropriate and consistent specimen collection across all sites. At minimum, the protocol or an external reference should include:*

- *Patient preparation*
- *Supplies needed*
- *Labeling Requirements*
- *Step-by-step collection instructions*
- *Minimal sample volume*
- *Storage requirements*
- *Specific clinical requirements (e.g., CSF must be collected by a properly trained and qualified clinician)*

*Minimum level of detail to include in the protocol or an external reference:* To avoid contamination with skin flora, strict aseptic procedures will be followed when collecting blood specimens. Initially, soap and clean water will be used to remove dirt/oils from the venipuncture site. Once the venipuncture site has been washed and dried it should be prepared by:

- Cleaning the venipuncture site with a sterile alcohol wipe (70% isopropanol or 70% ethanol), wiping in a concentric circle outward from the puncture site. When the alcohol dries, 10% povidone or 2% iodine tincture will be applied (also in a concentric circle outward from the puncture site). The iodine will be allowed to dry for 60 seconds prior to venipuncture. After sample collection, residual iodine will be removed using an alcohol wipe or clean water to avoid irritation.
- Cleaning the venipuncture site with a sterile, single use alcoholic-chlorhexidine scrub. The skin will be scrubbed in a concentric circle outward from the puncture site and allowed to dry for 30 seconds.

The vein will not be re-palpated without sterile gloves once the skin has been prepared with iodine or alcoholic chlorhexidine. In addition, the septum of the blood culture bottle will be cleaned with alcohol prior to inoculating the sample.

**Considerations for Adaptation**

Specimen collection: The protocol should include (or reference) protocols which provide an appropriate level of detail to ensure that all aspects of specimen collection are appropriate for the tests performed and consistent across all sites. The types of specimens collected will be largely dependent on the pathogens of interest and the diagnostic tests used to detect those pathogens. Blood is nearly always used for detecting a wide range of febrile etiologies, but additional samples (e.g., urine (clean catch method), NP/OP swabs) may be recommended to determine the system infected. It is usually simplest to collect the same set of samples from all patients. However, if resources are limited, alternatives include adding more samples in future expansions or collecting certain samples only from symptomatic patients (e.g., NP/OP swabs from those with respiratory symptoms). The trade-offs between sample sensitivity and feasibility of implementation will need to be weighed when deciding which specimen types to collect and from whom.

### Specimen Storage and Transport

*Specify parameters required to maintain integrity for each specimen type prior to testing including:*

- *Storage temperature and initial processing at site*
- *Time limits for sample processing (e.g., how long can a sample be held at X temperature prior to processing)*
- *Where samples will be stored prior to transport*
- *Transport requirements (temperature, packing, etc.)*

*Identify how and where any other specimens will be stored prior to testing, referencing* [best practices](https://apps.who.int/iris/bitstream/handle/10665/68554/WHO_CDS_CSR_RMD_2003.6_%28appendices%29.pdf?sequence=2&isAllowed=y) such as the [*Manual for Identification and Antimicrobial Susceptibility Testing (World Health Organization, 2003)*](https://apps.who.int/iris/bitstream/handle/10665/68554/WHO_CDS_CSR_RMD_2003.6_%28appendices%29.pdf?sequence=2&isAllowed=y)*. Describe the means and frequency of specimen transport from the location of specimen collection to the location of laboratory testing, including any intermediate locations.*

[DESIGNATED STAFF] will send the Specimen Requisition Form and the accompanying specimens for transport to [TESTING LOCATION]. During transportation, specimens will be stored in a cool box at [TEMPERATURE] until reaching [TESTING LOCATION]. Care will be taken with vacutainers as glass and plastic can leak and break.

### Specimen Processing and Testing

*Describe the laboratory testing procedures and complete Table 1 to summarize. Include, at minimum:*

- *List of pathogens to be tested*
- *Location(s) where laboratory testing will be performed*
- *Frequency of laboratory testing (e.g., daily, weekly), including time frame in which specific samples must be processed (e.g., urine samples must be plated within 24 hours)*
- *Specimen handling procedures*
- *Assays to be used on each sample type*
- *Quality control methods*
- *Use of standard operating procedures*
- *Interpretation of results*
- *Plans to conduct confirmatory testing or subtyping, if applicable*
- *Rejection criteria*

**Table 1: Laboratory Testing Procedures**

| **Specimen Type** | **Testing Method/Assay** | **Pathogen(s), Antigen(s), and/or Antibodies** | **Minimum Volume** |
| --- | --- | --- | --- |
| *Ex: whole blood, serum, NP/OP swab, urine, stool, sputum, tissue, CSF* | *Ex: RT-PCR, RDT, ELISA, IFA, culture* | *Ex: pathogen X RNA, pathogne X IgM antibody* | *Ex: 100 µl* |

**Considerations for Adaptation**

Laboratory Testing: The laboratory testing approach will vary considerably based on surveillance objectives, available resources, priority pathogens, existing testing platforms, and current knowledge about local AFI etiologies. Multiplex pathogen testing (e.g., TaqMan Array Card, BioFire) is often preferrable when countries are initiating AFI surveillance but can be costly to maintain. Countries often transition to pathogen-specific testing (e.g., PCR, culture) to achieve greater sustainability once the priority causes of AFI are known. Combinations of these tests for all or a subset of cases may also be considered.

### Specimen Archiving

*If applicable:* Specimens will be stored at -80° C for up to [NUMBER OF YEARS] for future testing. All future testing will occur at [LOCAL TESTING LOCATION] if capability exists. For tests that [LOCAL TESTING LOCATION] cannot perform, specimens will be shipped to [EXTERNAL TESTING LOCATION] for additional testing when feasible. Appropriate approvals (including specimen and materials transfer agreement if needed) will be obtained at the time of protocol approval.

**Considerations for Adaptation**

Secondary objectives: If specimens will be stored in a biobank for future testing, it is critical to consider the laboratory’s biosafety and biosecurity standards and procedures for procuring and maintaining freezers. Note that the informed consent (Section 5.1) will need to include the potential for future testing – otherwise, patients will need to be tracked down and reconsented for any additional testing.

# **Data Management and Analysis**

## **Data Management**

*Describe data management procedures. For each of the below activities, include the* ***location*** *(e.g., sentinel site, laboratory),* ***frequency*** *(e.g., daily, weekly), and* ***staff*** ***roles****.*

- *Data entry from paper forms (e.g., Case Report Form, Specimen Requisition Form) to electronic database and storage and archiving of paper forms, if applicable. For example:*

Every week, surveillance staff will enter data from the Case Report Form and Specimen Requisition Form into a digital database. After data entry, any paper versions of forms (Case Report Form, Specimen Requisition Form, Informed Consent Form) will be stored in locked long-term storage units at the sentinel sites for the duration of the project. Access to long-term storage will be restricted to appropriate personnel to review for management and supervisory purposes, data quality checks, and other operational aspects of the project.

- *Data upload from real-time electronic data collection tools (e.g., tablet, mobile device) to electronic database, if applicable. For example:*

Each day at each sentinel site, surveillance staff will download data from the electronic Case Report Form and conduct data transfers to the central database.

- *Electronic database management (e.g., software, internet connectivity). For example:*

Database managers will develop the digital database in Excel and provide troubleshooting and data quality checks as needed.

- *Linkage of laboratory results to electronic database using unique patient identifier. For example:*

Laboratory personnel will enter testing results into an encrypted and secured database every day. Linkage of demographic and epidemiologic data from the Case Report Form and laboratory results will occur passively from the electronic linkage of databases using the unique patient identification numbers.

- *Data back-ups to a central server. For example:*

Each day at each sentinel site, the database will be automatically backed up to a second secured server at a different geographic location than the primary project server. Each backup will make a complete copy of all data stored on the database. Once a week, a digital archive of the database will be recorded onto an external drive, which will be labeled appropriately and stored securely at a central location.

- *Data security measures (e.g., user access, confidentiality). For example:*

There will be restricted access to rooms housing tablet computers, laptops, desktop computers or systems containing surveillance data. Network access to surveillance data will be restricted to surveillance personnel. The database will be password-protected. Materials with identifiable information, such as informed consent and data collection forms, will be kept on computer servers with secure passwords and encrypted electronic storage devices. To protect patient privacy, only de-identified information will be entered into the digital dataset. Computers that contain or have access to surveillance data and connect to the internet will maintain updated anti-malware protection and use firewalls as appropriate.

- *Data quality control (e.g., audits, spot checks). For example:*

Each day at each sentinel site, the site supervisor will perform quality control by reviewing all completed forms for completeness and consistency. After transcription of study data into the database, data quality procedures will be performed to identify errors such as missing data, outlier date ranges, and validation checks; entries flagged as errors will be recorded and communicated to surveillance investigators for correction.

- *Data integration with national surveillance systems (e.g., LIMS, DHIS2), if applicable. For example:*

Laboratory personnel will enter testing results into the Laboratory Information Management System (LIMS). Unique identification numbers will be used to link the laboratory results to the epidemiologic and clinical information of the patient. Data will be extracted from LIMS and used for lab/epi data analysis.

## **Data Cleaning and Analysis**

Regular data cleaning and analysis will be performed by [PERSON(S) and/or INSTITUTIONS] using [SOFTWARE (E.G., SAS, SPSS, R, Excel, etc.)]*.* This will occur [FREQUENCY (e.g., weekly)] and reports will be distributed to [DESIGNATED PERSONNEL (e.g., PI, Co-PIs, Investigators)]. Data analysis will be primarily descriptive in nature, with a focus on *(including, but not limited to)*:

- Numbers, descriptions, and results of specimens tested
- Number of patients screened and enrolled
- Characteristics of enrolled AFI patients, including stratification by *(for example)*:
  - Age
  - Sex
  - Occupation
  - Education Level
  - Geography
  - Exposure

A more comprehensive report of surveillance findings will additionally be generated [FREQUENCY (annually at minimum)]. Development of the report should occur after laboratory testing has been finalized for the designated time frame of the report. This effort will be led by [PERSON(S) and/or INSTITUTIONS] using [SOFTWARE] and distributed to [DESIGNATED PERSONNEL].

*Describe how surveillance findings will inform any additional analyses and/or data products, as appropriate. Options may include:*

- *Development of scientific manuscripts and presentations*
- *Public health planning and policymaking*
- *Surveillance evaluations, including any measurements of direct and/or indirect costs*

**Considerations for Adaptation**

Data cleaning: Regular data cleaning allows for assessment of protocol implementation and progress. If data collection is paper-based, data must first be transferred to an electronic database before cleaning occurs. Data cleaning may include, but is not limited to, checking for and fixing incorrect formatting, incomplete responses, or duplicate entries.

Data analysis: Possibilities for data analysis vary depending on surveillance design, priority questions, and data elements collected. In the short term, data outputs can inform which changes, if any, need to be made to the surveillance protocol to improve the data being collected. This may be particularly useful in assessing which sites are underperforming and where additional resources may be needed. In the longer term, findings can help guide a wide range of scientific, programmatic, and clinical activities, depending on surveillance scope and context.

## **Data Sharing**

*Describe data ownership and sharing procedures as appropriate. If applicable, create a data sharing agreement detailing how surveillance data will be shared among investigators and partners.*

# **Ethical Considerations**

## **Informed Consent/Assent**

*Informed consent and assent procedures for surveillance participants will vary depending on country-specific context. An example description of informed consent procedures is provided below but should be adapted to national guidelines, surveillance specifics, and IRB requirements.*

Individuals who are eligible and who agree to participate will be asked to provide [WRITTEN and/or VERBAL] informed consent (for adults aged 18 years and older or emancipated minors aged 15–17 years) or [WRITTEN and/or VERBAL] parental permission followed by [WRITTEN and/or VERBAL] assent (for minors aged 15–17 years) using the procedures described in Section 3.3 (see Appendix [X] for informed consent/assent forms). Consent of parents or caregivers will be obtained for minors less than [NUMBER OF YEARS] old; assent from children under [NUMBER OF YEARS] old will not be sought.

If participant, minor, parent, or guardian is illiterate, the relevant informed consent, permission, or assent form(s) will be verbally read to them by surveillance staff in [LANGUAGE(S)]. The participant or parent or guardian will be requested to use a thumbprint in lieu of a signature in the presence of a witness who is not involved in this project.

The surveillance staff member will fill out and sign the informed consent form documenting the completion of consent procedures and marking enrollment in the surveillance; the case report form will thereafter be administered, and biological specimens will be collected from participants.

## **Risks**

*Risks for surveillance participants and personnel will vary depending on country-specific context. An example description of surveillance risks and serious adverse advents is provided below but should be adapted according to national guidelines, surveillance specifics, and IRB requirements.*

Enrollment in this surveillance presents no more than minimal risk to participants and refusal to participate will not in any way impact the care a patient receives at selected health facilities.

The only opportunity cost (no financial costs) to participants will be minor inconveniences resulting from the amount of time necessary to complete enrollment procedures, questionnaires, sample collection procedures, and return visits for the collection of convalescent samples, for which their travel costs will be reimbursed.

Drawing blood or other specimens can cause temporary discomfort, and rarely can lead to prolonged bleeding and bruising. Serious adverse events from these procedures are very rare. However, should a serious adverse event occur (e.g., excessive bleeding, infection at the phlebotomy site, etc.), the event will immediately be brought to the attention of the clinical staff on duty. The PI should be notified within 24 hours of the event and where necessary, the project will cover the medical expenses of affected participants.

Identifying information will be kept confidential and only used at the site level for the provision of clinical care and reporting of diagnostic laboratory tests to the treating clinician; this information will not be transmitted or used for any other reasons. Unique identification numbers assigned to all participants will be used to link questionnaire and laboratory data.

## **Benefits**

*Benefits for surveillance participants and personnel will vary depending on country-specific context. Examples of optional benefits are provided below.*

*Economic or other incentive to participants:*

Participants will be offered a small cash sum or equivalent incentive to compensate for inconvenience due to the time interviewing and blood draw.

*Economic incentive or improved skillset for surveillance personnel:*

For clinical personnel at sites involved in this project and performing duties above and beyond their routine clinical responsibilities (e.g., AFI patient screening, informed consent, specimen and data collection, etc.), a small monetary stipend compensation or equivalent will be given *(if allowable under surveillance and institutional rules and regulations)*. Other expected benefits include training on epidemiologic data collection and management and laboratory testing, and greater awareness of etiologies of febrile patients seen at their facilities.

*Use of test results for patient clinical care:*

Other than RDT results, in most cases, the enhanced testing performed will not provide a direct benefit to participants in the form of individual improved medical care, given the time delays expected in obtaining results from this off-site testing. However, participants may benefit individually from improved quality of care or patient management given increased overall medical provider knowledge of disease etiologies over time.

*Knowledge and capacity building applicable to the community and nation:*

The community may also benefit from improved disease control and prevention measures as a result of the expanded availability of laboratory diagnostics and knowledge of circulating pathogens. At the national level, improved surveillance capacity will improve communicable disease outbreak preparedness and response, as well as improve the availability of data for informing and targeting public health interventions and policies.

# **Timeline**

*Adapt the below table as needed to describe the surveillance timeline.*

|  | ***YEAR 1*** | | | | | | | | | | | | ***YEAR 2*** | | | | | | | | | | | | |
| --- | --- | --- | --- | --- | --- | --- | --- | --- | --- | --- | --- | --- | --- | --- | --- | --- | --- | --- | --- | --- | --- | --- | --- | --- | --- |
|  | ***J*** | ***F*** | ***M*** | ***A*** | ***M*** | ***J*** | ***J*** | ***A*** | ***S*** | ***O*** | ***N*** | ***D*** | ***J*** | ***F*** | ***M*** | ***A*** | ***M*** | ***J*** | ***J*** | ***A*** | ***S*** | ***O*** | ***N*** | ***D*** |  |
| **Protocol Development** |  |  |  |  |  |  |  |  |  |  |  |  |  |  |  |  |  |  |  |  |  |  |  |  |  |
| **Training** |  |  |  |  |  |  |  |  |  |  |  |  |  |  |  |  |  |  |  |  |  |  |  |  |  |
| **Enrollment** |  |  |  |  |  |  |  |  |  |  |  |  |  |  |  |  |  |  |  |  |  |  |  |  |  |
| **Data Analysis** |  |  |  |  |  |  |  |  |  |  |  |  |  |  |  |  |  |  |  |  |  |  |  |  |  |
| **Report Writing** |  |  |  |  |  |  |  |  |  |  |  |  |  |  |  |  |  |  |  |  |  |  |  |  |  |
| **ETC…** |  |  |  |  |  |  |  |  |  |  |  |  |  |  |  |  |  |  |  |  |  |  |  |  |  |

## **Future Protocol Adaptation**

*Recommended:* At the end of each project year, a technical panel, comprised of [KEY INVESTIGATORS AND ADVISORS (e.g., the principal investigator, co-principal investigator, technical advisors, the public health institute director)] will meet to review the AFI surveillance results and modify, as needed, the list of pathogens to continue testing. Furthermore, the panel will plan when and how, if needed, to change the surveillance laboratory testing platform to a more sustainable one. Investigators will submit the changes, as a protocol addendum, to the IRB for approval.
