# Supplementary material for: A toolkit for planning and implementing acute febrile illness (AFI) surveillance
Source: PLOS Glob Public Health. 2024 Apr 18;4(4):e0003115. doi: 10.1371/journal.pgph.0003115 (PMC11025857; doi:10.1371/journal.pgph.0003115)
Supplement: S5 File — (DOCX) [file pgph.0003115.s005.docx]

| Date of screening: _____ / _____ / __________ (DD/MM/YYYY) |
| --- |
| Screener name: ____________________________ , _________________________ (surname, first name) |
| Facility name: _________________________________ |

| **Screening Questions** | | |
| --- | --- | --- |
| 1) Previously enrolled in this Acute Febrile Illness surveillance? | | Yes  No |
| *If “yes,” stop completing form and mark patient as ineligible in question X.* | |  |
| *OPTIONAL (if case definition includes an age restriction):*  2) Age: ________ 2a) Age Units *(check one)*:  Years  Months  Day(s) | | |
| *If age is outside inclusion range, STOP: mark patient as ineligible in question X.* | | |
| 1. Current measured [AXILLARY, TYMPANIC, ORAL, AND/OR RECTAL] temperature ≥ [38°C]? | | Yes  No |
| *If “yes,” skip to question 5.* | | |
| *OPTIONAL (if case definition includes history of fever):*  4) History of fever in the last [7 days]? | | Yes  No |
| *If “no,” STOP: mark patient as ineligible in question X.* | | |
| 1. Fever onset date: _____ / _____ / _________ (DD/MM/YYYY) | | |
| *If fever onset occurred more than [7 days] ago, STOP: mark patient as ineligible in question X.* | | |
| 1. Presenting with a confirmed cause of fever? | | Yes  No |
| *If “yes,” STOP: mark patient as ineligible in question X.* | | |
| 1. Chief complaint on presentation to facility: | | |
| 7a) Injury or trauma?  Yes  No  7b) *OPTIONAL:* [ADDITIONAL SYNDROME(S) OUTSIDE SCOPE OF SURVEILLANCE]  Yes  No | | |
| *If “yes” to any of the above, STOP: mark patient as ineligible in question X.* | | |
| 1. Meets eligibility criteria? | | Yes  No |
| *If “no,” patient is ineligible for participation. Thank respondent for their time.* | | |
| *OPTIONAL (if sampling every nth patient):*  9) [Nth] eligible patient? | | Yes  No |
| *If “no,” patient will not be sampled for participation. Thank respondent for their time.*  *If “yes,” proceed to obtain informed consent or assent.* | | |
| 1. Patient consents or assents to enroll in this surveillance? | Yes  No | |
| *If “no,” thank respondent for their time.*  *If “yes,” proceed to Case Report Form.* | | |
