# Supplementary material for: A toolkit for planning and implementing acute febrile illness (AFI) surveillance
Source: PLOS Glob Public Health. 2024 Apr 18;4(4):e0003115. doi: 10.1371/journal.pgph.0003115 (PMC11025857; doi:10.1371/journal.pgph.0003115)
Supplement: S7 File — (DOCX) [file pgph.0003115.s007.docx]

| **Section I: Reporting Information** | | | | |
| --- | --- | --- | --- | --- |
| 1) *Unique patient ID: [ __ ] [ __ ] [ __ ] [ __ ] [ __ ] [ __ ] [ __ ] [ __ ] [ __ ] | 2) *Facility name: _____________________________ | | | |
| 3) *Date of interview: _____ / _____ / __________ (DD/MM/YYYY) | 4) *Interviewer name: _____________ , ____________ (surname, first name) | | | |
| **Section II: Patient Information** | | | | |
| 5) Patient name: ____________________________ , _________________________ (surname, first name) | | | | |
| 6a) Phone number: ____________________________________________ 6b) Alternate phone number (*optional*): ____________________________ | | | | |
| Residence Information (appropriate terminology to be filled into brackets – ex: province, region, county, district, community):  7a) [ADMIN LEVEL 1]: ____________ 7b) [ADMIN LEVEL 2]: ____________ 7c) [ADMIN LEVEL 3]: ____________ 7d) [ADMIN LEVEL 4]: ____________ | | | | |
| 8) *Date of birth: ____ /_____ /_____ (DD/MM/YYYY) *AND/OR* 9) *Age: ______ 9a) Age units *(check one)*:  Years  Months  Day(s) | | | | |
| 10) *Sex:  Female  Male  Other  *If female:* 10a) *Currently pregnant?  Yes  No  Unsure/prefer not to say | | | | |
| 11) *Currently hospitalized?  Yes  No *If yes:* 11a) Admission date: ___ /___ /______ (DD/MM/YYYY) | | | | |
| 12) *Temperature measured at the facility?  Yes  No *If no, skip to question 13.*  12a) *Maximum recorded temperature: ________ °C  12b) *Measurement mode of maximum temperature:  Axillary  Oral  Rectal  Tympanic  Other method | | | | |
| **Section III: Course of Illness** | | | | |
| 13) *When did your fever start? (approximately) _____ / _____ / __________ (DD/MM/YYYY) | | | | |
| 14) Was temperature measured before arriving to this facility?  *If yes:* 14a) Was measured temperature ever greater than or equal to 38°C? | | | Yes  No  Unsure  Yes  No  Unsure | |
| 15) Did you take any fever-reducing medications (ex: paracetamol, aspirin,  non-steroidal anti-inflammatory drugs) in the last 8 hours? | | Yes  No  Unsure | | |
| 16) Did you take any antimicrobial medications (ex: antibiotics, antimalarials)  in the last 72 hours? | | Yes  No  Unsure | | |
| 17) When did you experience the first symptom(s) of this illness? (approximately) _____ / _____ / __________ (DD/MM/YYYY) | | | | |
| **18) *Within the past 14 days (including today), did you experience any of the following signs or symptoms?** | | | | |
| 18a) Chills or rigors | | | | Yes  No  Unsure |
| 18b) Muscle or joint pain (myalgia or arthralgia) | | | | Yes  No  Unsure |
| 18c) Abdominal pain | | | | Yes  No  Unsure |
| 18d) Nausea or vomiting | | | | Yes  No  Unsure |
| 18e) Diarrhea (≥3 loose stools/24hrs) | | | | Yes  No  Unsure |
| 18f) Rash | | | | Yes  No  Unsure |
| 18g) Headache | | | | Yes  No  Unsure |
| 18h) Runny nose (rhinorrhea) | | | | Yes  No  Unsure |
| 18i) Sore throat | | | | Yes  No  Unsure |
| 18j) Cough | | | | Yes  No  Unsure |
| 18k) Shortness of breath (dyspnea) | | | | Yes  No  Unsure |
| 18l) Chest pain | | | | Yes  No  Unsure |
| 18m) Loss of taste | | | | Yes  No  Unsure |
| 18n) Loss of smell | | | | Yes  No  Unsure |
| 18o) Other, specify: ____________________ | | | | Yes  No  Unsure |

*Implementers to decide if/how they would like to include the* ***setting-specific questions*** *below on their AFI Surveillance Case Report Form, depending on the specifics of their AFI surveillance activities. Option to REMOVE or ADD additional questions or answer choices that are of interest.*

| **Section II: Patient Information** | |
| --- | --- |
| **Additional demographic Information** | |
| Marital Status:  Single  Married or living together  Prefer not to say  Divorced, separated, or widowed | Race/Ethnicity/Nationality:  [Option 1]  [Option 3]  [Option 5]  [Option 2]  [Option 4]  [Option 6] |
| Highest education received:  No education  Below primary  Secondary  Prefer not to say  Primary  Above secondary | Occupation:  [Option 1]  [Option 4]  [Option 7]  [Option 10]  [Option 2]  [Option 5]  [Option 8]  [Option 11]  [Option 3]  [Option 6]  [Option 9]  [Option 12] |
| **Vaccine History: Has the patient ever received any of the following vaccines?** | |
| Rotavirus  Rubella  Yellow Fever  *Haemophilus influenzae* Type b (Hib)  Pneumococcal Conjugate  Meningitis  Typhoid  Dengue Fever  Hepatitis B  Measles  Influenza  Polio  Japanese Encephalitis  Diphtheria, Tetanus, and Pertussis (DTP)  COVID-19  *If vaccinated for COVID-19:*  Vaccine Brand Name *(check one)*: Number of doses and dates administered *(check all that apply)*:  Pfizer-BioNTech  Moderna  Johnson & Johnson’s Janssen  Dose 1 Date: ____________  AstraZeneca  Sinovac  Sinopharm  Dose 2 Date: ____________  Other, specify: _____________ | |
| **Patient Follow-Up & Outcome: to complete if surveillance activities include patient follow-up** | |
| *If hospitalized*: Discharge date (DD/MM/YYYY): ___ /___ /______  Was the outcome of this illness fatal?  Yes  No  Unknown outcome  Clinical diagnosis by healthcare provider* (*check all that apply*):  Unexplained fever  [Illness]  [Illness]  [Illness]   [Illness]  [Illness]  [Illness]  [Illness]  [Illness]  [Illness]  [Illness]  [Illness]  [Illness]  [Illness]  [Illness]  Other illness, specify: ____________ | |

| **Section III: Course of Illness** |
| --- |
| **Additional Facility Testing** |
| Was the patient tested for malaria for this illness?  Yes  No  Unsure  If yes, which test(s) and what was/were the result(s)? (*check all that apply)*?  Rapid Diagnostic Test:  Positive  Negative  Unknown result  Microscopy:  Positive  Negative  Unknown result  Was the patient tested for COVID-19 for this illness?  Yes  No  Unsure  If yes, which test(s) and what was/were the result(s)? (*check all that apply)*?  Rapid test:  Positive  Negative  Unknown result  PCR:  Positive  Negative  Unknown result |

| **Section IV: Exposure Information** | | | | | | | | | | | |
| --- | --- | --- | --- | --- | --- | --- | --- | --- | --- | --- | --- |
| **Animal Exposures** | | | | | | | | | | | |
| In the month before the patient’s illness, did he/she: | If yes, type of animal (*check all that apply)* | | | | | | | | | | |
|  | Dogs/  Cats | Poultry | Birds, not including poultry | Cattle | | Goats/  sheep | Pigs | Rodents | Monkeys | Bats | Other, specify:  ___________ |
| eat any animals?  Yes  No  Unsure  If yes, specify the animals: |  |  |  |  | |  |  |  |  |  |  |
| touch/handle any animals?  Yes  No  Unsure  If yes, specify the animals: |  |  |  |  | |  |  |  |  |  |  |
| hunt/slaughter any animals?  Yes  No  Unsure  If yes, specify the animals: |  |  |  |  | |  |  |  |  |  |  |
| help birth/deliver any animals?  Yes  No  Unsure  If yes, specify the animals: |  |  |  |  | |  |  |  |  |  |  |
| sheer/tan any animals?  Yes  No  Unsure  If yes, specify the animals: |  |  |  |  | |  |  |  |  |  |  |
| In the month before illness, was the patient bitten by any ticks?  Yes  No  Unsure  In the month before illness, was the patient bitten by any animal?  Yes  No  Unsure  If yes, specify type of animal: __________________  In the month before illness, did the patient eat, sell, handle or have any other form of contact with bushmeat?  Yes  No  Unsure  If yes, please describe: ___________________________________________________________________________ | | | | | | | | | | | |
| **Environmental and Other Exposures**  In the month before the patient’s illness, has the patient had contact with any of the following? (*check all that apply)* | | | | | | | | | | | |
| Drinking untreated water?  Yes  No  Unsure | | | | | Contact with other ill person(s)  Yes  No  Unsure | | | | | | |
| [Other exposure]  Yes  No  Unsure | | | | | Consumption of raw/undercooked meat?  Yes  No  Unsure | | | | | | |
| [Other exposure]  Yes  No  Unsure | | | | | [Other exposure]  Yes  No  Unsure | | | | | | |
| **Travel History**  In the month before the patient’s illness, has the patient travelled outside of country?  Yes  No  Unsure | | | | | | | | | | | |
| **Country** | | | | | **Dates of Travel (*approximately) DD/MM/YYYY*** | | | | | | |
|  | | | | | ___ / ___ / ______ − ___ / ___ / ______ | | | | | | |
|  | | | | | ___ / ___ / ______ − ___ / ___ / ______ | | | | | | |
|  | | | | | ___ / ___ / ______ − ___ / ___ / ______ | | | | | | |
