# Supplementary material for: A toolkit for planning and implementing acute febrile illness (AFI) surveillance
Source: PLOS Glob Public Health. 2024 Apr 18;4(4):e0003115. doi: 10.1371/journal.pgph.0003115 (PMC11025857; doi:10.1371/journal.pgph.0003115)
Supplement: S8 File — (DOCX) [file pgph.0003115.s008.docx]

| **Section I: Specimen Collection and Transport** |
| --- |
| 1) *Unique patient ID: [ __ ] [ __ ] [ __ ] [ __ ] [ __ ] [ __ ] [ __ ] [ __ ] [ __ ] |
| 2) Patient name: ____________________________ , _________________________ (surname, first name) |
| 3) Originating facility: _________________________________ |
| 4) Specimen collector name: ____________________________ , _________________________ (surname, first name) |
| 5a) *Date of specimen collection: _____ / _____ / __________ (DD/MM/YYYY) 5b) Time of specimen collection: _____:______ (HH:MM) |
| 6) *Whole blood collected?  Yes  No  *If not collected, specify reason*: ______________________________________________________ |
| 7) *Serum collected?  Yes  No  *If not collected, specify reason*: ______________________________________________________ |
| 8) *[SAMPLE X] collected?  Yes  No  *If not collected, specify reason*: ______________________________________________________ |
| 9a) Date of specimen transport: _____ / _____ / __________ (DD/MM/YYYY) 9b) Time of specimen transport: _____:______ (HH:MM) |

| **Section II: Laboratory Testing** | | | | | |
| --- | --- | --- | --- | --- | --- |
| 10) Testing facility: ____________________________________ | | | | | |
| 11) Name of person receiving specimen(s): ____________________________ , _________________________ (surname, first name) | | | | | |
| 12a) Date specimen(s) received: _____ / _____ / __________ (DD/MM/YYYY) 12b) Time specimen(s) received: _____:______ (HH:MM) | | | | | |
| **13) Specimen processing:** | | | | | |
| **Sample Type** | ***Sample Condition** | | **Number of Aliquots** | | **Notes** |
| Whole blood | Accepted  Rejected  *If rejected, specify reason*: _______________________ | | # Processed: ______  # Stored: ______ | |  |
| Serum | Accepted  Rejected  *If rejected, specify reason*: _______________________ | | # Processed: ______  # Stored: ______ | | Hemolytic  Lipemic |
| [SAMPLE X] | Accepted  Rejected  *If rejected, specify reason*: _______________________ | | # Processed: ______  # Stored: ______ | |  |
| **14) *Laboratory test results:** | | | | | |
| **Laboratory Test/Platform** | **Date of Test** | **Pathogens Tested** | | **Test Results** | |
| [TEST X] | _____ / _____ / ______ (DD/MM/YYYY) | [PATHOGEN X] | | Positive (+)  Negative (-)  Inconclusive | |
|  |  | [PATHOGEN X] | | Positive (+)  Negative (-)  Inconclusive | |
|  |  | [PATHOGEN X] | | Positive (+)  Negative (-)  Inconclusive | |
|  |  | [PATHOGEN X] | | Positive (+)  Negative (-)  Inconclusive | |
|  |  | [PATHOGEN X] | | Positive (+)  Negative (-)  Inconclusive | |
|  |  | [PATHOGEN X] | | Positive (+)  Negative (-)  Inconclusive | |
|  |  | [PATHOGEN X] | | Positive (+)  Negative (-)  Inconclusive | |
| [TEST X] | _____ / _____ / ________ (DD/MM/YYYY) | [PATHOGEN X] | | Positive (+)  Negative (-)  Inconclusive | |
|  |  | [PATHOGEN X] | | Positive (+)  Negative (-)  Inconclusive | |
| [TEST X] | _____ / _____ / ________ (DD/MM/YYYY) | [PATHOGEN X] | | Positive (+)  Negative (-)  Inconclusive | |
| [TEST X] | _____ / _____ / ________ (DD/MM/YYYY) | [PATHOGEN X] | | Positive (+)  Negative (-)  Inconclusive | |
