# Supplementary material for: A toolkit for planning and implementing acute febrile illness (AFI) surveillance
Source: PLOS Glob Public Health. 2024 Apr 18;4(4):e0003115. doi: 10.1371/journal.pgph.0003115 (PMC11025857; doi:10.1371/journal.pgph.0003115)
Supplement: S9 File — (DOCX) [file pgph.0003115.s009.docx]

*Informed consent and assent procedures for surveillance participants will vary depending on country-specific context. Template informed consent and assent forms are provided below but should be adapted according to national guidelines, surveillance specifics, and IRB requirements.*

| **ADULT CONSENT TO PARTICIPATE IN AFI SURVEILLANCE** |
| --- |

**(Note: this consent form is required to be read, explained, and signed/agreed to by all participants age >=18 years and by parents or guardians of participants age <18 years)**

The [IMPLEMENTING ORGANIZATION] is conducting surveillance to find out why people in [COUNTRY] develop acute febrile illness (fever). We are asking volunteers of all ages presenting to a health facility with symptoms of acute febrile illness to answer a short survey, have their blood drawn [AND OTHER SAMPLES COLLECTED (if applicable)] by [DESIGNATED STAFF], and get tested to see why they are sick.

We would like you to be part of this surveillance project. Your participation in the project is voluntary. You can choose whether to be in the project. Before you decide, we would like to tell you about the surveillance and what it would be like to be to participate. We will answer any questions you have.

**Purpose of the surveillance**

We will collect blood [AND ADDITIONAL SAMPLE TYPES (if applicable)] from patients in this facility. Those samples will be tested to help us better understand how febrile illness spreads. The surveillance will help us find out how they may have been exposed.

**What will it involve?**

If you agree to participate, we will ask you questions about your health and your recent activities, and we will draw about 5-10mls of blood through a needle in your arm. *If applicable:* If you agree, we will also take a throat swab by brushing a small cotton swab on the back of your throat or in your nose.

In total, it will take about 15 minutes. We will test the samples to see what germs are causing the acute febrile illness recently or in the past. We may also send your samples out of the country in accordance with the law for further testing that cannot be performed in [COUNTRY] and for quality assurance; this will only occur with the permission of the government of [COUNTRY]. You are free to change your mind if you do not want your samples to be stored for further testing. If you do not want your samples to be stored/examined for future use/other testing, the samples will be destroyed after all testing is completed for this surveillance. As part of a future evaluation, we may also contact you in the future. At that time, you may decide if you want to be part of the evaluation or not. You may also decline if you do not want to be contacted.

**Cost**

There is no cost to you. The testing is free.

**Benefits**

There is no direct benefit to you as a participant, but this surveillance may benefit this health facility, your community, and the [COUNTRY] government over time. The results will give us information that could help prevent people from getting sick and improve care for people with fever attending this health facility. *If applicable:* You will be offered a [SMALL CASH SUM OR EQUIVALENT INCENTIVE] to compensate for your time participating in this surveillance activity.

**Risks**

The only risk is that you may have some moderate, brief pain or stinging sensation when we take blood from your arm. A small bruise may also appear. All the people who draw blood for this survey are fully trained in doing so. They will do everything they can do to decrease your pain.

**Confidentiality**

Your name and information will be kept private to the full extent possible. We will keep all sample forms and results in locked file cabinets and only surveillance staff and doctors will be allowed to look at them. When we talk or write about this surveillance, we will not include your name or other facts that might identify you. The results of the acute febrile illness tests are confidential. We will not tell anyone else what your test results are. You may be contacted in case of an emergency.

**Future Contact**

*If applicable:* We may also conduct an evaluation of this project. It is your choice whether to be contacted for a project evaluation and whether to participate. Agreeing to be contacted does not mean that you must participate in the evaluation.

**For more information**

We are happy to answer any questions or concerns about the surveillance. If you have any questions about the project, please contact [NAME AND CONTACT INFORMATION OF PI].

**Voluntary Participation**

It is your choice whether to be in this project. You will still receive all necessary medical attention even if you decide not to be in the project and you can choose to stop at any time.

**If you agree to participate, please sign or make your mark below:**

Do you agree for us to take your blood to test for what is causing your fever? □ Yes □ No

*If applicable:*

Do you agree for us to swab your nose and throat to test for what is causing your fever? □ Yes □ No

Do you agree to the storage of your samples for future testing? □ Yes □ No

Do you agree to being contacted in the future for a possible evaluation? □ Yes □ No

**Name of Participant (printed):** __________________________________________________________

**Signature or Fingerprint of Participant:** ___________________________________________________

**Name of Participant’s Parent/Witness (printed):** ___________________________________________

**Signature or Fingerprint of Participant’s Parent/Witness**: ____________________________________

**Date**: ____/____/________

*dd mm yyyy*

| **CHILD ASSENT TO PARTICIPATE IN AFI SURVEILLANCE** |
| --- |

**(Note: This child assent form is required to be read, explained, and signed/agreed to by participants ages 8-17 years)**

We are conducting surveillance to find out why people get sick with acute febrile illness (fever), which can be caused by many different things. We would like you to be in this project. You don’t have to be a part of our project unless you want to.

**What will happen?**

If you accept to participate, we will take a small amount of blood (up to 5mls) from you by putting a needle in your arm for a few seconds*. If applicable:* If you agree, we will also take a throat swab by brushing a small cotton swab on the back of your throat or in your nose.

**Will it hurt?**

The needle stick in your skin may hurt a little for a few seconds.

**Why are we doing this surveillance?**

The tests are being done for this surveillance. It you let us take samples from you, it will help us understand why you have fever.

**You can say no**

You can say no, and we won’t do the tests. You will not be in trouble if you say no.

**If you agree to participate, please sign or make your mark below:**

Do you agree for us to take your blood to test for what is causing your fever? □ Yes □ No

*If applicable:*

Do you agree for us to swab your nose and throat to test for what is causing your fever? □ Yes □ No

**Name of Participant (printed):** __________________________________________________________

**Signature or Fingerprint of Participant:** ___________________________________________________

**Date**: ____/____/________

*dd mm yyyy*
