# Supplementary material for: A toolkit for planning and implementing acute febrile illness (AFI) surveillance
Source: PLOS Glob Public Health. 2024 Apr 18;4(4):e0003115. doi: 10.1371/journal.pgph.0003115 (PMC11025857; doi:10.1371/journal.pgph.0003115)
Supplement: Attachment — Submitted filename: Response to reviewers.pdf [file pgph.0003115.s010.pdf]

A Toolkit for Planning and Implementing Acute Febrile Illness (AFI) Surveillance
Manuscript: PGPH-D-23-01745
PLOS Global Public Health

January 26, 2024

Dear Dr. Muñiz-Salazar,

We appreciate the thoughtful feedback provided by the reviewers on our manuscript. We are grateful for the opportunity to address these suggestions and resubmit the revised manuscript for further consideration.

We recognize the importance of the primary recommendation from the reviewers to demonstrate the toolkit's effectiveness and applicability in real-world scenarios. In fact, our primary motivation for pursuing publication is to disseminate the toolkit so that it may be utilized and improved based on feedback received from practical applications. Unfortunately, it was not possible for us to implement a true "pilot study" given funding limitations. While some CDC partner countries have made use of components of the toolkit, we do not have field data from a context that has adapted the toolkit in its entirety. In addition, we believe that true validation will be difficult due to the inherent flexibility of the toolkit, which leads to great variability in adaptation and implementation. We hope that the incorporation of lessons learned from years of experience supporting AFI surveillance, in addition to the interviews conducted as part of the evaluation process, provide sufficient evidence of the practical utility of the toolkit.

In line with the valuable suggestion to improve the manuscript's clarity, we have developed a figure illustrating the toolkit's components and their interrelationships. We hope that this visual aid will contribute to a clearer presentation of the toolkit's structure and intended use.

Below this letter, we have included point-by-point responses to the reviewers' individual questions and concerns. We have attempted to address each comment and believe that the revisions have enhanced the quality of this work.

We appreciate your time and consideration and welcome any additional feedback.

Thank you,

Lilit Kazazian, MS
Health Scientist, Division of Global Health Protection
U.S. Centers for Disease Control and Prevention

Reviewer #1

In the manuscript "A toolkit for planning and implementing acute febrile illness (AFI) surveillance" by Lilit Kazazian and colleagues, submitted to PLOS Global Public Health, the authors developed the toolkit for planning and implementing Acute febrile illness (AFI) surveillance. The toolkit provides a comprehensive framework to guide researchers and public health officials, to identify and/or monitor the potential causes of AFI. The toolkit includes a set of planning aids and supporting materials, an implementation framework, generic protocol, several generic forms (including screening, case report, specimen collection and testing, and informed consent and assent), and a generic data dictionary. The authors claim these materials incorporate key elements intended to harmonize approaches for AFI surveillance, as well as setting-specific components and considerations for adaptation based on local surveillance objectives and limitations. Finally, the authors suggest that the appropriate adaptation and implementation of the toolkit may generate data that expand the global AFI knowledge base, strengthen countries' surveillance and laboratory capacity, and enhance outbreak detection and response efforts.

The study was well conducted, the documents generated are comprehensive and carefully produced, and the manuscript is clear and well presented. The value of the documents presented here are of great relevance and should be adopted and implemented in relevant settings.

Author response: Thank you!

Concerns and comments:

1) The toolkit was developed with the input of many experts. However, was the toolkit ever tested in a pitot study or target population? Is there some field data that could be presented here as an example of the application of the toolkit?

Author response: Thank you for raising this concern. We share the reviewer's sentiments and explain why a pilot study was not conducted in the above letter.

2) When applying the toolkit in a proper setting, large amount of data will be generated. Despite the suggestion of using DHIS2 or LIMS, is there a program developed or being developed specifically to receive data coming from the toolkit forms and analyze it fast and efficiently?

Author response: As noted in lines 209-212, we developed the DHIS2 AFI module in collaboration with the University of Oslo as an extension to the toolkit. This module includes a data entry platform and a dashboard for analyzing AFI surveillance data in real-time. Just as the toolkit itself, the DHIS2 module is generic, and both the data entry platform and dashboard would need to be modified based on the protocol and forms used in specific contexts. However, we intentionally avoid recommending any single program for AFI surveillance, as this decision is ultimately dependent on context-specific factors.

3) For the sake of simplicity, please include a workflow diagram describing all the documents of the toolkit, including the set of planning aids and supporting materials, the implementation framework, generic protocol, generic forms (including screening, case report, specimen collection and testing, and informed consent and assent), and a generic data dictionary.

Author response: Thank you for this valuable suggestion. We have developed a workflow diagram as described to improve clarity.

Reviewer #2

The toolkit provides a flexible framework for researchers and public health officials to plan and implement AFI surveillance. It includes an implementation framework, a generic protocol, forms, and a data dictionary, aiming to harmonize approaches and adapt to local needs. It emphasizes the need for methodological standardization while accommodating different approaches and goals.

I think this will be very helpful and I can imagine recommending this to students/colleagues.

Author response: Thank you! We are encouraged by this feedback.

I did when initially opening the paper think that it was going to be a very different paper with an algorithm and workflow for particular testing and contexts, including more on microbiology decisions. So it may help to clarify this more in the title and abstract. I thought halfway through the abstract this might be a mini lab approach to fever.

Author response: Thank you for sharing this perspective. We were unsure about how to improve clarity and consulted with laboratorian colleagues who did not share the reviewer's interpretation of the title and abstract. We would welcome any specific suggestions on how to avoid confusion.

I think this can be accepted as it is now and have said this separately as well, but it would be improved with a refinement of the who/what/when/where/why sections. Most of the explanations are just listing of information. Giving perhaps a pull out example of particular cases and how it is used would help or going into more depth, succinctly, of how the context and population were determined to affect the use of the tool.

Author response: Thank you for this feedback. We believe that the workflow diagram developed in response to the suggestion by Reviewer #1 will help to demonstrate how its components can be applied at various stages of planning and implementation. We hope that this addition addresses this reviewer's concerns as well.
